# Exploring entrepreneurial intention among European Union youth by education and employment status

**DOI:** 10.1371/journal.pone.0318001

**Published:** 2025-01-24

**Authors:** Maria Denisa Vasilescu, Elen-Silvana Crivoi, Andreea-Monica Munteanu

**Affiliations:** 1 Department of Wages, Income and Taxation, National Scientific Research Institute for Labour and Social Protection, Bucharest, Romania; 2 Department of Statistics and Econometrics, Bucharest University of Economic Studies, Bucharest, Romania; 3 Faculty of Sociology and Social Work, University of Bucharest, Bucharest, Romania; Alexandru Ioan Cuza University: Universitatea Alexandru Ioan Cuza, ROMANIA

## Abstract

The relatively high vulnerability of young Europeans in the labour market compared to other age groups has led many to seek alternative employment solutions, such as entrepreneurship. While not a comprehensive solution, entrepreneurial initiatives among youth can offer a valuable opportunity for their integration into stable and decent work. This research uses Flash Eurobarometer 513—Social Entrepreneurship and Youth to explore entrepreneurial intentions among European Union youth. The study’s novelty consists of the division of the target population into four distinct categories based on educational and employment status. The results indicate that EU youth are not a homogeneous group. While the motivation to create their own jobs—is a common feature of all young individuals with entrepreneurial intentions, suggesting a necessity-driven entrepreneurial intention, significant differences emerge among sub-groups. Young people who are only studying exhibit an interest in exploiting business opportunities, highlighting the role of education in fostering opportunity-driven entrepreneurship, while NEETs and employed youth are primarily motivated by better income prospects in the intention to start a business. Additionally, youth engaged in education and those integrated into the labour market are better informed about financing and training opportunities Perceived barriers include a lack of education for NEETs and financial risks for others. Funding preferences also differ: NEETs would rely on personal savings, while those balancing study and work would explore diverse sources, including venture capital and incubator support for starting businesses. Our study enhances the understanding of entrepreneurial intentions among EU young people, offering insights that can guide more effective support strategies for fostering entrepreneurial success.

## Introduction

European youth are in a vulnerable position within the labour market, characterized by disproportionately high unemployment rates compared to other demographic groups. In 2023, the youth unemployment rate within the European Union (EU) stood at 11.2%, with Greece and Spain reporting rates exceeding 20% [1]. In contrast, the overall unemployment rate was approximately 6% during the same period [2]. The rate of young people not in employment, education, or training (NEETs) mirrored the youth unemployment rate at 11.2%, with countries such as Romania experiencing NEET rates exceeding 19% in 2023 [3].

Entrepreneurship may offer a potential pathway for the labour market integration of young people and can enhance their sense of purpose in life [4]. Although businesses established by young entrepreneurs are generally less likely to employ a large workforce compared to those created by older entrepreneurs, they provide significant opportunities for self-employment. Many young people are driven to start their own businesses due to limited job opportunities [5]. With adequate policy support, young people can transition from low-wage jobs or low-profit self-employment to creative and innovative entrepreneurship that offers decent working conditions [6].

European Union youth are people aged between 15–30 years old, of any nationality of a European Union country, that live in one of the 27 EU Member States [7].”Entrepreneurship is an individual’s creative capacity, independently or within an organisation, to identify an opportunity and to pursue it in order to produce new value or economic success” [8]. Entrepreneurial intention is”the conscious state of mind that precedes action towards a goal such as starting a new business” [9].

The literature on youth entrepreneurship [10] identifies a multitude of traits of young people who engage in business activities, including specific attitudes and expectations, skills and education, access to support infrastructure such as financial and training opportunities, familiarity with entrepreneurship, and socio-demographics such as gender and place of residence. However, the research also reveals gaps and contradictory findings. For instance, while some studies [11] have identified a link between entrepreneurial attitudes and the intention to start a business, others [12] have found no such association. Regarding entrepreneurship education, while a lack of education is recognized as a significant barrier to youth business involvement [13], other studies [14] suggest that entrepreneurial training may raise awareness but does not necessarily increase the intention to start a business among young people. Familiarity with entrepreneurship tends to enhance youth interest in business [15,16], although working in family businesses appears to reduce their entrepreneurial interest [17]. Regarding gender, although young men generally exhibit higher entrepreneurial intentions than females, in certain regions of Europe, gender seems to not influence entrepreneurial intentions [18].

Moreover, the papers [10–17] tend to study specific categories of youth, the most frequent categories being high school and university students. Another studied category consists of young people not in education, employment, or training [19,20]. Other papers [21,22] compare between European youth from different countries. Using the same database as our research (Flash Eurobarometer 513—Social Entrepreneurship and Youth), a recent study [21] focuses on the comparison between Romanian and EU youth’s intentions of becoming entrepreneurs. However, comparisons between European Union youth based both on their employment and educational status are scarce, contrary to the initiatives of international organizations that draw attention to the fact that young people are not a homogeneous group [23].

Consequently, the aim of this research is to identify the characteristics of EU youth with entrepreneurial intentions, differentiating between four sub-groups of young people aged 15–30 in the European Union: NEETs, young Europeans who are only studying, young Europeans who are only working, and young Europeans who both study and work. Building on the gaps identified in the literature, the main research question is ‘How are young people with entrepreneurial intentions from each of the four sub-groups characterized in terms of their attitudes, expectations, skills, education, familiarity with entrepreneurship, and socio-demographic factors?”. The hypotheses of our research are: H1: Within the four analysed sub-groups, young Europeans with positive attitudes toward entrepreneurs are statistically more inclined to have entrepreneurial intentions; H2: Within the four analysed sub-groups, young Europeans who have positive expected outcomes in relation to entrepreneurship are statistically more inclined to have entrepreneurial intentions; H3: Within the four analysed sub-groups, young Europeans who lack entrepreneurship skills are statistically less inclined to have entrepreneurial intentions; H4: Within the four analysed sub-groups, young Europeans who lack financial resources are statistically less inclined to have entrepreneurial intentions; H5: Within the four analysed sub-groups, young Europeans familiar with entrepreneurship are statistically more inclined to have entrepreneurial intentions; H6: Within the four analysed sub-groups, young European males are statistically more inclined to have entrepreneurial intentions compared to women; H7: Within the four analysed sub-groups, young Europeans under 25 years old are statistically more inclined to have entrepreneurial intentions compared to young Europeans aged 25 and older; H8: Within the four analysed sub-groups, young Europeans living in urban areas are statistically more inclined to have entrepreneurial intentions compared to those living in rural areas.

The novelty of our research consists of the analysis of four distinct sub-groups within the population of young Europeans. Given the assumption that young people in Europe are not a homogeneous group [24], we divide the general population of interest into four groups based on their educational and employment statuses: NEETs, young people who are solely studying, young people who are solely working, and those balancing work with study. We seek to identify both the common features shared by all young people and the specific characteristics of each sub-group in relation to their entrepreneurial intentions.

This article is structured as follows: the Literature review section documents the existing research on entrepreneurial intentions among young people, with a focus on European youth, while identifying key gaps in the literature. The Materials and methods section details the data sources and methodology, emphasizing the use of the Flash Eurobarometer 513—Social Entrepreneurship and Youth. It also explains the application of logistic regression models to analyse how young individuals with entrepreneurial intentions from four distinct groups are characterized based on various factors, such as opinions toward entrepreneurship, expected outcomes of entrepreneurial initiatives, and sources of information on entrepreneurship. In the Results and discussion section, we analyse how young Europeans who are studying and working, only studying, only working, or classified as NEETs are distinguished according to these key variables. The implications of these findings for youth entrepreneurship policy are also discussed. Finally, the Conclusions section compares the four groups, offering insights based on the analysis of European youth entrepreneurship intentions.

## Literature review

Young people with entrepreneurial intentions are characterized by specific features, including personal traits, family characteristics, and factors related to both community and society. Numerous studies examine the barriers and facilitators of entrepreneurship among youth, particularly for students from Europe, Asia, Australia, the United States, and Africa [25].

Attitudes are defined as”a relatively stable system of beliefs concerning some object, that result in an evaluation of that object” [26]. The relationship between attitudes and intention is supported by the Theory of Planned Behavior, which suggests that attitudes–essentially, an individual’s positive or negative evaluation of entrepreneurship–along with subjective norms and perceived behavioral control are the key antecedents of the intention to become an entrepreneur [27]. Consistent with this theory, a study [11] on 1,200 economics and business students from four South-East European countries (Bosnia and Herzegovina, Croatia, North Macedonia, and Serbia) found a positive relationship between attitudes and entrepreneurial intentions. Using an extensive set of explanatory variables, a study [10] among students in Serbia aims to predict entrepreneurial intentions and conclude that "attitudes positively affect students’ intentions to start their own business." Similarly, personal attitudes toward entrepreneurship significantly influence the intention to start a business among youth in South America and Asia [28,29]. Additionally, a study involving students from Iran, Poland, Germany, India, Spain, and the Netherlands underscores the importance of positive attitudes toward entrepreneurship in shaping the intention to start a business [9]. A cross-cultural comparison between Türkiye and the Republic of Kosovo [30] finds that personal attitudes positively moderate the relationship between the need for achievement and both entrepreneurial intention and alertness. Similarly, a study among university students in Poland and Spain [31] concludes that subjective factors, such as beliefs, social norms, and values influence entrepreneurial attitudes. These attitudes, in turn, translate into action drivers of entrepreneurship, including the intention to start a business. However, a study on Polish students [12] found that positive attitudes toward entrepreneurial activities did not significantly influence the reported intention to start a business. In our study, we aim to examine the relationship between opinions and cognitive attitudes regarding entrepreneurship and the intention to start a business among individuals aged 15–30 in the European Union, and identify how these opinions and attitudes differ between the four studied sub-groups if the case.

H1: *Within the four analysed sub-groups*, *young Europeans with positive attitudes toward entrepreneurs are statistically more inclined to have entrepreneurial intentions*.

Certain personality traits are commonly observed among young aspiring entrepreneurs. Traits such as the need for autonomy and achievement, ambition, creativity, innovation, openness to experience, risk-taking, and proactivity are positively correlated with entrepreneurial intentions and actions among youth [32]. In Slovakia, entrepreneurial intention among high school students is associated with leadership, autonomy, academic interest, and a proactive personality [33]. Similarly, in Montenegro, young individuals engaged in business exhibit a strong desire for change and independence [34]. In low-income countries such as Malaysia, willingness to take risks and creativity are significant factors influencing youth involvement in business, often outweighing the impact of educational background and training [35]. However, not all entrepreneurial intentions are motivated by factors such as ambition and creativity. The necessity to find a job and to earn more are entrepreneurship motivators among youth [36]. While “opportunity” entrepreneurship is pro-cyclical and more oriented to growth, “necessity” entrepreneurship is countercyclical, a solution to unemployment [37]. In our research, we aim to test how expected outcomes such as creating jobs for themselves or the others, accomplishing personal growth, following passions and opportunities etc. relate to the entrepreneurial attitudes, and what are the differences and similarities between the four sub-groups in this regard.

H2: *Within the four analysed sub-groups*, *young Europeans who have positive expected outcomes in relation to entrepreneurship are statistically more inclined to have entrepreneurial intentions*.

A substantial body of literature directly links skills and education to entrepreneurship. A theoretical paper [38] describes how entrepreneurial education positively influences business development. In Poland, young people perceive the lack of entrepreneurial education as a significant barrier to business activities [13]. Young entrepreneurs in the Western Balkans face several changes including inadequate integration of entrepreneurial education with existing curricula, insufficient methods and equipment such as ICT infrastructure, and outdated skills among educators [36]. A study [39] involving 1,150 university students from 55 universities in China concludes that formal entrepreneurial education enhances opportunity recognition, thereby increasing entrepreneurial intention. The study also finds that the relationship between education and opportunity recognition is strengthened by individual learning and self-development in entrepreneurship. However, another study [33] found that entrepreneurial education, which focuses on the transmission of skills and knowledge, does not affect high school students’ entrepreneurial intentions. In contrast, entrepreneurial training, which emphasizes the transmission of motives and attitudes is beneficial primarily for young people living in rural areas. Similar findings regarding the impact of education on entrepreneurship have been reported among students in Serbia, although the results vary depending on the statistical methods employed [10].

But education is linked to entrepreneurship indirectly also. The perception of self-efficacy and perceived behavioural control–concepts related to the self-assessment of one’s capabilities to perform entrepreneurial tasks–encourages business activity [40–42]. Self-efficacy predicts the intention to start a business, with this relationship being further strengthened by education, a study [43] involving 334 Indian students confirms. Concurrently, fear of failure acts as a deterrent to entrepreneurial intentions among youth. A comparative study between Greece and Germany [44] found that fear of failure negatively impacts business ownership, with its prevalence and intensity varying across analysed countries.

However, experimental research on Jordanian middle and high school students found that while a specific financial literacy program increased participants’ awareness of entrepreneurship, it did not positively affect their intention to start a business [14]. Consequently, we aim to assess whether youth in the EU perceive education and skills as barriers to starting a business and identify the preferred sources of entrepreneurial training each of the four sub-groups of youth rely on (e.g., school and university, social media, etc.).

H3: *Within the four analysed sub-groups*, *young Europeans who lack entrepreneurship skills are statistically less inclined to have entrepreneurial intentions*.

Accessing funding is a major barrier for young entrepreneurs, a challenge that has intensified since the 2008 financial crisis [5]. Research in Europe underscores the critical role of financial access in shaping youth entrepreneurship intentions. Specifically, young people in Montenegro and Serbia have highlighted financial access as a significant challenge in entrepreneurship [34,45]. Theoretical papers addressing developing and emerging economies [32] or more specific regions like the Western Balkans [36] further emphasize the importance of a supportive financial environment, including the availability of grants, debt financing, and equity financing. In Romania, young entrepreneurs face difficulties such as high loan interest rates, limited leasing options, and currency instability [46,47]. Research from low-income countries such as Tanzania, Ghana, and Nepal ([48] highlights the lack of personal or family funds as an important barrier, emphasizing the crucial role of access to financial support. Nevertheless, when studying the link between entrepreneurial intention and subsequent startup activities, another study [49] found that while the quality of legal institutions significantly moderates the relationship between intention and reaction, no significant association was found between the national financial system and the intention-action relationship. Among financial sources, equity financing, such as funds from family and friends, can be crucial for young aspiring entrepreneurs [50]. In the northeastern region of Romania, students rely on the availability of family and friends’ funds when considering starting a business [47]. Our research aims to evaluate the impact of financial resources and financial risks on the decision of European youth to start a business and to identify the primary sources of financing each of the four sub-groups consider.

H4: *Within the four analysed sub-groups*, *young Europeans who lack financial resources are statistically less inclined to have entrepreneurial intentions*.

The entrepreneurial background of family and friends appears to play a crucial role in shaping the intention of youth to start a business. In Germany, knowing other business founders encourages young people to pursue entrepreneurship [42]. In Spain, family interest in business and the support provided by families positively influence youth entrepreneurial intentions [15,16]. Similarly, in Ghana, young people from entrepreneurial families are more likely to have the intention to become entrepreneurs themselves [51], while in Pakistan, young people considering an entrepreneurial career place considerable importance on having relatives who own a business [52]. Another study [53] found that among Chileans under 30, a background in family business and a sense of self-fulfillment significantly contribute to the intention to start a business in the near future. However, some research presents a more nuanced view. For example, Greek students with less than one year of experience in a family business showed reduced intention to engage in entrepreneurship during times of crisis [17]. Additionally, a study [54] on 130 management students from Mumbai found no significant differences in the intention to start a business between students from family business backgrounds and those from salaried backgrounds. Consequently, our research aims to examine the relationship between familiarity with entrepreneurship and the intention to start a business within the four sub-groups of young Europeans.

H5: *Within the four analysed sub-groups*, *young Europeans familiar with entrepreneurship are statistically more inclined to have entrepreneurial intentions*.

Young men generally exhibit a higher intention to start a business compared to women. Specifically, among economics and business students in four Southeast European countries, young women have a lower entrepreneurial intention [11]. Similarly, entrepreneurial activities are more prevalent among young men than their female counterparts [42]. A study on Finnish students [55] noted a disparity in attitudes among female students: while approximately 70% believe young people are interested in entrepreneurship, fewer than 60% intend to start a business themselves. However, other studies present a contrasting view. For instance, research conducted at a university in the Netherlands indicates that students are equally interested in pursuing innovative economic activities regardless of gender, with status-seeking and risk-taking identified as more important drivers of entrepreneurship [18]. The influence of age on entrepreneurial intention is less frequently explored in the literature, suggesting that individuals aged 15 to 30 may not be a homogeneous group. Some authors [10] found a positive correlation between age and the intention of young Serbian students to engage in entrepreneurial activities. Consequently, we aim to investigate whether there are differences in entrepreneurial intention based on gender and/or age within the four groups of young individuals in the European Union.

H6: *Within the four analysed sub-groups*, *young European males are statistically more inclined to have entrepreneurial intentions compared to women*.
H7: *Within the four analysed sub-groups*, *young Europeans under 25 years old are statistically more inclined to have entrepreneurial intentions compared to young Europeans aged 25 and older*.

A study involving 1,497 Finnish students aged 15 to 24 years found that regional factors significantly influence entrepreneurial intention. Specifically, students from more developed areas with tertiary education institutions, a robust industry, and a prevalence of white-collar jobs exhibited higher intentions to start a business compared to those from less developed regions [55]. Similarly, among 537 Slovak high school graduates, those residing in cities had a higher intention to start a business than their counterparts from towns [33]. This disparity may be attributed to the greater availability of business and learning opportunities in more developed regions compared to less affluent areas. Nevertheless, other research [56] found no correlation between the place of residence (rural or urban) and the entrepreneurial intentions of Serbian students in tourism and hospitality management. Therefore, we aim to examine whether there are significant differences in entrepreneurial intentions within the four sub-groups of youth in the European Union based on their place of residence.

H8: *Within the four analysed sub-groups*, *young Europeans living in urban areas are statistically more inclined to have entrepreneurial intentions compared to those living in rural areas*.

Although numerous studies examined youth entrepreneurial intention, distinguishing between different subgroups of young people based on education and employment status is relatively uncommon. In this context, the novelty of our research lies in categorizing young people in the European Union into four relevant subgroups and analysing them separately: NEETs, those who are solely studying, those who are solely working, and those who are both studying and working.

## Materials and methods

The analysis conducted in this research uses data from the Flash Eurobarometer 513—Social Entrepreneurship and Youth. The data were collected in October 2022, based on a web-based self-administered questionnaire, by Ipsos European Public Affairs [7]. The survey targeted young residents of the EU member states, aged between 15 and 30 years. The sample includes 25,984 individuals, with around 500 respondents from Luxembourg, Cyprus, and Malta, and approximately 1000 young people from each of the other EU27 member states. The sample is weighted according to the proportion of 15–30 year-old population for each analysed country.

The dataset employed to explore youth entrepreneurial intentions included a range of socio-demographic characteristics, such as age, gender, residential environment, family composition, and education level. Furthermore, variables reflecting young people’s opinions toward entrepreneurship were incorporated. These included reasons for starting a business, perceived barriers, important goals, preferred sources of initial financing, favoured providers of entrepreneurship education, types of support deemed important, and general opinions about entrepreneurs. The research items were organized in alignment with both our conceptual framework and the structure of the Eurobarometer questionnaire. A detailed description of all variables can be found in Table 1.

**Table 1:** Description of variables.

Variable	Description and coding	
Entrepreneurship intention	Would you consider setting up your own business? 0 = No 1 = Yes	
Gender	Gender of the respondent: 0 = Female, 1 = Male	
Age	Age of the respondent: 0 = 15–24 years, 1 = 25–30 years	
Type of community	Residential environment: 1 = Rural area or village, 2 = Small or medium-sized town, 3 = Large town/city	
Household size, 15+	Number of people aged 15 years or more living in the household, including the respondent: 1 = One person; 2 = Two persons; 3 = Three persons; 4 = Four or more persons aged 15 or more	
Number of children under 15 years	Number of children under the age of 15 living in the household: 1 = No children; 2 = One child; 3 = Two children; 4 = Three or more children	
Level of formal education	***For young people who are still studying***: 1 = Lower secondary education 2 = Upper secondary level, general education 3 = Upper secondary level, vocational education and training 4 = Post‐secondary, non‐higher education 5 = Higher education	***For the youth who have finished education***: 1 = Left school before completing lower secondary education 2 = Lower secondary education 3 = Upper secondary level, general education 4 = Upper secondary level, vocational education and training 5 = Post‐secondary, non‐higher education 6 = Higher education
Familiarity with entrepreneurship	How familiar are you with entrepreneurship? 0 = Not very much or Not at all; 1 = A great deal or A fair amount	
Young people’s interest in entrepreneurship	In your view, how interested are people of your age in starting their own business? 0 = Not very interested or Not at all interested; 1 = Very interested or Fairly interested	
Opinions/cognitive attitudes	Which of the following statements are closest to your opinion? Coding: 0 = No; 1 = Yes ➢ Entrepreneurs create new products and services that benefit us all ➢ Entrepreneurs only think about profit ➢ Entrepreneurs aim for a better world ➢ Entrepreneurs make a lot of money ➢ Entrepreneurs are job creators	
Expected outcomes	Which of the following reasons do you think drive young people to start their own business? Coding: 0 = No; 1 = Yes ➢ Freedom to choose time and place of work ➢ Better income prospects ➢ Exploiting a business opportunity ➢ Lack of employment opportunities If you were to set up your own business, which of the following goals would be most important to you? Coding: 0 = No; 1 = Yes ➢ To become wealthy ➢ To create my own job ➢ To follow my passion/interest ➢ To create jobs for others ➢ To put innovative ideas into practice Which of the following reasons, do you think stop young people from becoming entrepreneurs? Coding: 0 = No; 1 = Yes ➢ Financial risks involved ➢ Access to social protection (health insurance and unemployment benefits) ➢ Concerns about red tape and administrative issues (e.g. accounting obligations, tax returns) ➢ Concerns about workload, working hours and/or responsibilities ➢ Unfavourable economic climate	
Specific skills and education	Which of the following reasons, do you think stop young people from becoming entrepreneurs? Coding: 0 = No; 1 = Yes ➢ Lack of knowledge/education/skills on how to start and run a business If you were to set up your own business, what type of support would be the most useful? Coding: 0 = No; 1 = Yes ➢ Support with developing the business plan ➢ Support with tax requirements ➢ Education or training	
Financial resources	Which of the following reasons, do you think stop young people from becoming entrepreneurs? Coding: 0 = No; 1 = Yes ➢ Not enough capital/resources to be self-employedIf you were to set up your own business, what type of support would be the most useful? Coding: 0 = No; 1 = Yes ➢ Financial support in the form of grants ➢ Financial support in the form of conventional loans ➢ Microfinance support (e.g. microcredits, microloans, microinsurance)	
Initial funding sources	If you were to set up your own business, where would you seek the initial funding? Coding: 0 = No; 1 = Yes ➢ My own savings ➢ Family and/or friends ➢ Banks ➢ Microfinance providers ➢ Venture capital investments ➢ Business incubator ➢ My current salary	
Entrepreneurship training	If you were to set up your own business, which of the following options would you seek for entrepreneurship training? Coding: 0 = No; 1 = Yes ➢ School or university ➢ Entrepreneurship coach and network ➢ Online content creators (e.g. YouTube, Instagram, TikTok) ➢ Chamber of commerce ➢ Job centre ➢ EU institutions	
EU effort to decrease youth unemployment	How much effort do you feel the European Union is putting into decreasing youth unemployment? 0 = Not very much effort or No effort at all 1 = A great deal of effort or Some effort	

Given that the European youth population is not a homogenous group, we opted to analyse the target population separately, using two key dimensions, participation in education and employment, that define the younger generation. Based on these dimensions, we divided young people in the European Union into four subgroups: NEETs (not in education, employment, or training), those who are only studying, those who are only working, and those who are both studying and working.

To achieve our objective of identifying the profiles of young individuals with entrepreneurial intentions, we employed binary logistic regression. This approach facilitates the identification of distinct patterns, enabling the examination of similarities and differences across the four youth groups categorized by their participation in education and employment. By adopting this framework, we aim to generate deeper insights into the entrepreneurial aspirations within the European youth population.

We used multivariate binary logistic regression to assess the likelihood of the outcome variable being classified into one of two categories based on multiple explanatory variables. The dependent variable, denoted as Y, can take two values: 0 for failure (i.e., the respondent does not consider starting a business) and 1 for success (i.e., the respondent is considering starting a business). This model, known as the logit model, is based on conditional probabilities [57].

Let *P* be the probability associated with a successful event conditioned by multiple independent variables, generally noted X_p,_ where $p=\bar{1,m}$.


$$P=E(Y=1|X_{1},X_{2},\ldots ,X_{m})=\frac{1}{1+e^{-(\alpha +\beta_{1}*X_{1}+\beta_{2}*X_{2}+\cdots +\beta_{m}*X_{m})}}$$ (1)

The simplified form of the Eq (1) frequently used in practice is:

$$P=E(Y=1|X_{1},X_{2},\ldots ,X_{m})=\frac{1}{1+e^{-Z}}$$ (2)

Contrary, 1−*P* represents the probability of an unsuccessful event to occur. Based on Eq (2) the probability of failure is the following:

$$1-P=\frac{1}{1+e^{Z}}$$ (3)

The odds ratio is a measure widely used to illustrate the chances of a favourable event to happen:

$$\frac{P}{1-P}=e^{Z}$$ (4)

Applying the natural logarithm on the Eq (4) leads to obtaining the logit model:

$$Z=\ln\frac{P}{1-P}=\alpha +\beta_{1}*X_{1}+\beta_{2}*X_{2}+\cdots +\beta_{m}*X_{m}$$ (5)

The coefficients of the logit model are estimated using the maximum likelihood method.

In the general form of the model (Eq 5), the *X*_*m*_ terms denote the independent variables included in the analysis, such as socio-demographic characteristics and entrepreneurial attitudes. The intercept is represented by *α* and the coefficients *β*_*m*_ indicate the magnitude and direction of the relationship between each predictor and the likelihood of entrepreneurial intention.

Building upon the conceptual framework grounded in the literature, the analysis carried out in this study followed several steps. Initially, we examined entrepreneurial intentions across the entire sample, followed by a focused analysis of the four specific groups. Subsequently, using descriptive analysis, we provided a concise overview of the socio-demographic characteristics relevant to each of the four groups under investigation. Next, we employed the Chi-square test to examine the association between entrepreneurial intentions and the variables that describe the characteristics and opinions of young people (see S1 Table). The research was further advanced by estimating four separate logistic regression models, each corresponding to one of the four distinct youth groups, with the aim of identifying the unique profile of individuals exhibiting entrepreneurial intentions. This approach allows for a nuanced analysis that accounts for differences in occupational status and labour market engagement, offering a more tailored understanding of how different characteristics shape entrepreneurial aspirations within each subgroup.

## Results and discussions

**Fig 1:**
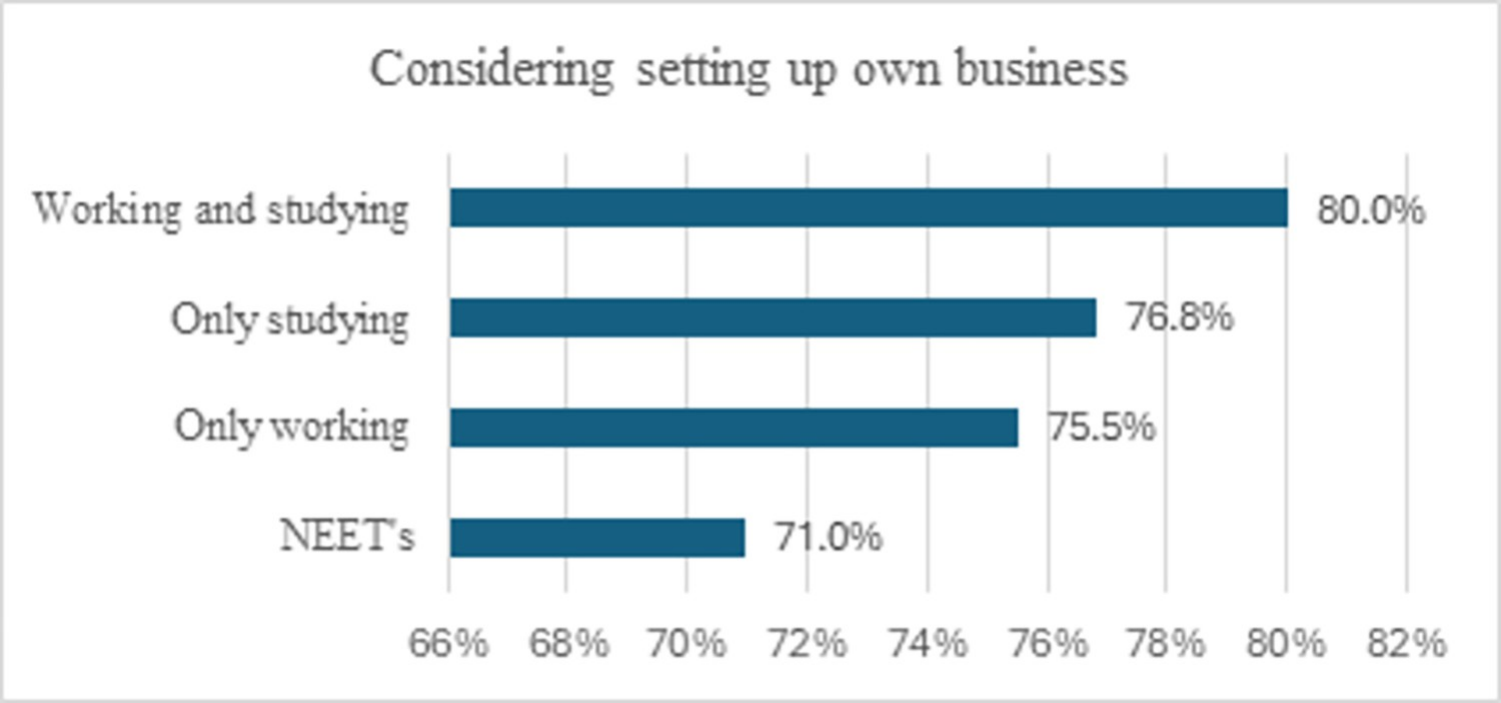
The share of young people who intend to become entrepreneurs, depending on their educational and labour market status. Source: Authors calculations.

Across the entire sample, 77.2% of young people aged 15 to 30 declared an intention to start their own business. However, when examining entrepreneurial intentions within the four distinct subgroups of youth, notable disparities emerged. The group most interested in entrepreneurship was young individuals who work and study, with 80% of them stating that they plan to start a business (see Fig 1). This group might exhibit the highest entrepreneurial intentions, as they benefit from both educational development and work experience, providing a strong foundation for entrepreneurial aspirations. Both young people who are only studying (76.8%) and those who are only working (75.5%) exhibit a high interest in entrepreneurship. Educational environments might be cultivating entrepreneurial thinking through exposure to new ideas and innovation, while working individuals may be gaining practical insights and opportunities that inspire entrepreneurial ambitions. NEET youth register the lowest percentage (71%), which is still an important proportion when it comes to their goal to become entrepreneurs. It is possible that this group has the lowest share of entrepreneurial intentions, as being disengaged from both work and education may limit their exposure to entrepreneurial opportunities or skills development. Moreover, the Chi-square test of independence revealed a statistically significant association between entrepreneurial intentions and educational and employment status.

To facilitate a more detailed exploration of the similarities and differences among European youth, we computed descriptive statistics to provide a clear summary of the dataset’s key features (see Table 2), with a particular focus on the socio-demographic characteristics of the four groups of young people. The NEET group has a higher proportion of women, a larger number of children within the household, and a lower educational level. In contrast, the studying group tends to be younger, residing in households with a greater number of adults. The working group comprises slightly older individuals, predominantly living in larger cities, part of smaller households, and with relatively higher levels of education. Finally, the studying and working group is characterized by a higher proportion of men, mostly residing in urban areas.

**Table 2:** Descriptive statistics.

	Gender	Age groups	Type of community	Household size, 15+	Household size, < 15	Education level
Young individuals not in education, employment, or training						
N	2684	2715	2715	2250	2308	2600
Minimum	0	1	1	1	1	1
Maximum	1	2	3	4	4	6
Mean	0.36	1.46	1.90	2.40	1.79	3.93
Std. Deviation	0.48	0.50	0.75	1.05	0.93	1.51
Young individuals who are only studying						
N	5251	5320	5320	4358	4512	5146
Minimum	0	1	1	1	1	1
Maximum	1	2	3	4	4	5
Mean	0.50	1.12	1.99	2.70	1.52	3.56
Std. Deviation	0.50	0.32	0.75	1.13	0.81	1.41
Young individuals who are only working						
N	6632	6660	6660	5932	6007	6595
Minimum	0	1	1	1	1	1
Maximum	1	2	3	4	4	6
Mean	0.45	1.69	2.08	2.20	1.45	4.71
Std. Deviation	0.50	0.46	0.77	0.98	0.76	1.39
Young individuals who are both studying and working						
N	10903	10971	10971	9310	9500	10575
Minimum	0	1	1	1	1	1
Maximum	1	2	3	4	4	5
Mean	0.51	1.41	2.03	2.33	1.56	3.78
Std. Deviation	0.50	0.49	0.76	1.06	0.83	1.33

Source: Authors calculations.

### Young NEETs

The econometric estimation results concerning the entrepreneurial intentions of young individuals who are neither employed nor enrolled in educational programs (NEETs) are presented in Table 3. The findings facilitated the outline of a sociodemographic profile of young individuals who intend to open their own businesses. Young men are more likely to manifest entrepreneurial intentions compared to young women. In terms of age, young people aged between 15–24 years are more likely to want to start their own business, compared to young adults (25–30 years). NEET young people who live in small or medium-sized towns are 33% more likely than those from rural areas to show entrepreneurial intentions. The results are consistent with other studies on youth [58] that identified a greater prevalence of having a business idea, among men, `older`youth, and youth in urban areas compared to their counterparts.

**Table 3:** Estimation results for young NEETs.

Variable	Coefficient	Odds ratio
Gender (Female—ref)	0.379*	1.461
Age (15–24 years—ref)	-0.221***	0.801
Type of community (Rural area or village—ref)		
*Small or medium-sized town*	0.286**	1.331
*Large town/city*	0.054	1.056
Number of children under 15 years (No children—ref)		
*1 child*	0.305**	1.357
*2 children*	0.308***	1.361
*3 or more children*	0.243	1.275
Highest level of education completed (Left school before completing lower secondary education—*ref*)		
*Lower secondary education*	0.718**	2.052
*Upper secondary level*, *general education*	0.521***	1.684
*Upper secondary level*, *vocational education*	0.463***	1.588
*Post‐secondary*, *non‐higher education*	0.452	1.571
*Higher education*	0.266	1.305
Familiarity with entrepreneurship	0.813*	2.256
Young people’s interest in entrepreneurship	0.569*	1.766
Reasons to start own business—Better income prospects	0.414*	1.513
Barriers to becoming an entrepreneur—Lack of knowledge/education/skills on how to start and run a business	0.242**	1.274
Barriers to becoming an entrepreneur—Concerns about administrative issues	0.259**	1.296
Important goals—To become wealthy	-0.340*	0.712
Important goals—To create my own job	0.591*	1.805
Important goals—To follow my passion/interest	0.224**	1.251
Important goals—To put innovative ideas into practice	0.429*	1.536
Initial funding sources—My own savings	0.474*	1.607
Entrepreneurship training—School or university	-0.497*	0.609
Entrepreneurship training—Online content creators (e.g. YouTube, Instagram, TikTok)	0.379*	1.460
Support perceived useful—Support with developing the business plan	0.242***	1.274
Support perceived useful—Microfinance support (e.g. microcredits, microloans, microinsurance)	0.420**	1.523
General opinion—Entrepreneurs create new products and services that benefit us all	0.288**	1.333
General opinion—Entrepreneurs only think about profit	-0.292**	0.747
Constant	-1.288*	0.276
*Number of observations*	*1943*	
*Omnibus Tests of Model Coefficients (Chi-square)*	*252*.25*	
*Nagelkerke R Square*	*0*.*175*	
*Hosmer and Lemeshow Test*	*Sig*. *= 0*.*909*	
*Overall percentage of classification table (accuracy)*	*74%*	

Note

* - 1% significance level

** - 5% significance level

*** - 10% significance level.

Source: Authors estimations.

Upon analysing the household composition of these young individuals, the results revealed no significant differences in entrepreneurial intentions based on the number of adults in the household. However, the number of children present in the household proved to be statistically significant. Specifically, young individuals from households with one or two children demonstrated a higher likelihood of aspiring to start their own businesses compared to those from households without children.

We also examined the potential impact of educational attainment on NEET youth’s propensity to pursue entrepreneurship. The results of the logistic regression indicated that young graduates of lower secondary education are twice as likely to want to open a business as those who left school before completing lower secondary education. Young individuals who have graduated from upper secondary education programs, whether general or vocational, exhibit a higher propensity for entrepreneurial aspirations compared to those who exited the educational system before completing lower secondary education. For higher levels of education (post-secondary and higher education), the results are not statistically significant.

In terms of beliefs and perceptions, familiarity with entrepreneurship emerges as a crucial factor affecting the entrepreneurial intentions of young NEETs. Those who possess knowledge and understanding of entrepreneurship are significantly more likely to plan to pursue this career route. Furthermore, youths’ intentions to start a business are influenced by the general belief that young people have a strong interest in entrepreneurship.

Young NEETs are more likely to aspire to become entrepreneurs themselves if they believe that having better income prospects is the primary motivator for young people between the ages of 15 and 30 to start a business. Regarding perceived obstacles to starting a business, young NEETs who are more inclined toward entrepreneurship commonly identify the main barriers as a lack of knowledge, education, and skills required for starting and managing a business, along with administrative challenges.

The findings also showed that young NEETs who are more prone to entrepreneurship have as important goals to create their own jobs, follow their passion or interest, or put innovative ideas into practice. An aspect worth emphasizing is that young individuals who perceive that becoming wealthy is the goal of entrepreneurship are less likely to express a desire to start their own business.

Regarding the initial sources of financing for the businesses that young people wish to start, the results indicate a strong preference for utilizing personal savings. No other financing option has been shown to be statistically significant in relation to the entrepreneurial intentions of young NEETs.

The analysis of the relationship between the desire to start a business and the preferred entrepreneurship training options for young NEETs revealed notable findings. Those who choose to learn about entrepreneurship via online content creators–such as through platforms like YouTube, Instagram, and TikTok–demonstrate a greater likelihood of intending to start their own business. However, those who learn about entrepreneurship in school or at university are less likely to wish to start their own company. This suggests that formal education may not be perceived by young people as effective in fostering an entrepreneurial spirit or providing the essential knowledge required to launch and manage a business.

Young NEETs who declared that they would consider it useful for starting a business to receive support for the development of the business plan and microfinance support, such as microcredits, microloans, and microinsurance, are more likely to have entrepreneurial intentions.

Young individuals who perceive entrepreneurs as creators of new products and services that benefit society are more inclined to want to start their own businesses. On the other hand, those who view entrepreneurs as solely profit-driven are less likely to aspire to become entrepreneurs themselves.

Although many initiatives focus on integrating NEETs into the labour market through entrepreneurship [59,60], the literature on NEETs drivers and facilitators in entrepreneurship is scarce, which reinforces the usefulness of our study.

### Young people who are only studying

Table 4 presents the results of the logistic regression analysis conducted to identify the factors influencing the entrepreneurial intentions of young individuals who are exclusively studying. The results once again indicate that young men who are only studying are more likely than women to aspire to start their own businesses. In terms of age, no significant differences were observed between young people aged 15–24 and those aged 25–30. Compared to individuals from families without children, young people from households with one child are more likely to have entrepreneurial intentions. Regarding the current level of education, young individuals enrolled in upper secondary education, whether general or vocational, are more likely to aspire to become entrepreneurs compared to those currently in lower secondary education. For post-secondary and tertiary education, the coefficients are not statistically significant.

**Table 4:** Estimation results for young people who are only studying.

Variable	Coefficient	Odds ratio
Gender (Female—ref)	0.277*	1.319
Age (15–24 years—ref)	-0.077	0.926
Type of community (Rural area or village—ref)		
*Small or medium-sized town*	0.020	1.020
*Large town/city*	-0.095	0.909
Number of children under 15 years (No children—ref)		
*1 child*	0.417*	1.517
*2 children*	0.032	1.032
*3 or more children*	0.264	1.303
Current level of education (Lower secondary level—ref)		
*Upper secondary level*, *general education*	0.377***	1.458
*Upper secondary level*, *vocational education*	0.372***	1.451
*Post‐secondary*, *non‐higher education*	0.194	1.215
*Higher education*	0.123	1.131
Familiarity with entrepreneurship	0.630*	1.878
Young people’s interest in entrepreneurship	0.469*	1.598
Reasons to start own business—Exploiting a business opportunity	0.275**	1.316
Barriers to becoming an entrepreneur—Financial risks involved	-0.197**	0.821
Barriers to becoming an entrepreneur—Concerns about workload, working hours and/or responsibilities	-0.429*	0.651
Barriers to becoming an entrepreneur—Unfavourable economic climate	-0.468*	0.626
Important goals—To create my own job	0.384*	1.468
Important goals—To put innovative ideas into practice	0.179***	1.196
Initial funding sources—My own savings	0.499*	1.648
Initial funding sources—Banks	-0.319*	0.727
Initial funding sources—Microfinance providers	0.323***	1.382
Initial funding sources—Venture capital investments	0.404**	1.498
Entrepreneurship training—School or university	-0.304*	0.738
Entrepreneurship training—Entrepreneurship coach or network	0.319*	1.375
Entrepreneurship training—Online content creators (e.g. YouTube, Instagram, TikTok)	0.450*	1.568
Entrepreneurship training—Job centre	-0.261**	0.770
General opinion—Entrepreneurs create new products and services that benefit us all	0.346*	1.413
General opinion—Entrepreneurs aim for a better world	0.549*	1.732
General opinion—Entrepreneurs make a lot of money	0.205***	1.227
EU is putting effort into decreasing youth unemployment	-0.219**	0.803
Constant	0.084	1.088
*Number of observations*	*3673*	
*Omnibus Tests of Model Coefficients (Chi-square)*	*375*.94*	
*Nagelkerke R Square*	*0*.*149*	
*Hosmer and Lemeshow Test*	*Sig*. *= 0*.*091*	
*Overall percentage of classification table (accuracy)*	*78*.*2%*	

Note

* - 1% significance level

** - 5% significance level

*** - 10% significance level.

Source: Authors estimations.

Familiarity with entrepreneurship emerges as one of the strongest determinants of entrepreneurial intentions among young individuals who are still enrolled in education. Furthermore, the belief that youth are generally interested in entrepreneurship raises the likelihood that a young person may wish to launch a company. The results are consistent with other research that targeted specific European countries such as Spain [15,16].

In terms of perceptions and opinions, young individuals who consider a business opportunity a reason to start a business are more likely to exhibit entrepreneurial intentions. On the other hand, young people who perceive financial risk, unfavourable economic climate, or concerns related to workload, working hours, and responsibilities as barriers to entrepreneurship are less likely to express a desire to start their own companies, pointing to the importance of the expected negative outcomes for the intention to start a business among youth who is only studying. These findings regarding financial risk and the importance of financial infrastructure are consistent with other studies among students across nine countries [49].

Among young individuals who are still in education, significant entrepreneurial goals include creating their own employment opportunities and implementing innovative ideas. Those who prioritize these objectives are more likely to have strong entrepreneurial intentions.

Regarding the initial financing sources, the entrepreneurial intentions of young individuals currently pursuing their studies are associated with options such as personal savings, microfinance providers, and venture capital investments. This reflects a preference for a more diverse range of financing methods. A significant finding is the negative coefficient associated with banks, suggesting a potential lack of confidence among aspiring young entrepreneurs in traditional financial institutions.

Young individuals who prefer to learn about entrepreneurship through online content creators on platforms such as YouTube, Instagram, and TikTok, as well as those who choose to engage with an entrepreneurship coach or network, are more likely to express an intention to start their own business. In contrast, those who learn about entrepreneurship at job centers and schools or universities are less likely to have entrepreneurial intentions.

The general perceptions of entrepreneurs held by young individuals who are still studying, which are associated with a higher likelihood of developing entrepreneurial intentions, include the views that entrepreneurs create new products and services that benefit society, aim for a better world, and make a lot of money. The importance of attitudes related to entrepreneurship for the intention to start a business is confirmed by numerous studies on students [10,11].

The study also examined the perceptions regarding the European Union’s efforts to reduce youth unemployment. Young individuals who believe that the EU is actively working to address youth unemployment are less likely to express an intention to start their own business. This finding suggests that those who have confidence in the EU’s efforts to enhance labour market opportunities for young people may be more inclined toward traditional employment rather than entrepreneurship. The study of attitudes toward the EU and their influence on entrepreneurial intentions is less explored, with findings indicating a predominance of necessity-driven motivations for starting a business.

### Young people who are only working

An additional group of young individuals, specifically those who are working but not studying, was analysed separately. The results of the econometric estimation for this group are presented in Table 5. Young men are more likely than young women to have entrepreneurial intentions. Additionally, individuals aged 25 to 30 are less likely to express a desire to develop their own businesses compared to those under 25 years old. The residential environment did not emerge as a significant determinant of entrepreneurial intentions among young workers. Concerning household composition, the number of adults does not appear to be a significant factor. Instead, young individuals from households with two or more children are more likely to express the desire to start a business.

**Table 5:** Estimation results for young people who are only working.

Variable	Coefficient	Odds ratio
Gender (Female—ref)	0.592*	1.808
Age (15–24 years—ref)	-0.276*	0.759
Type of community (Rural area or village—ref)		
*Small or medium-sized town*	-0.074	0.929
*Large town/city*	-0.011	0.989
Number of children under 15 years (No children—ref)		
*1 child*	0.119	1.126
*2 children*	0.331**	1.392
*3 or more children*	0.463**	1.589
Highest level of education completed (Left school before completing lower secondary education—ref)		
*Lower secondary education*	-0.533	0.587
*Upper secondary level*, *general education*	-0.720***	0.487
*Upper secondary level*, *vocational education*	-0.715***	0.489
*Post‐secondary*, *non‐higher education*	-0.667	0.513
*Higher education*	-0.919**	0.399
Familiarity with entrepreneurship	1.152*	3.166
Young people’s interest in entrepreneurship	0.643*	1.902
Reasons to start own business—Freedom to choose time and place of work	0.154**	1.166
Reasons to start own business—Better income prospects	0.389*	1.476
Barriers to becoming an entrepreneur—Financial risks involved	-0.338*	0.713
Barriers to becoming an entrepreneur—Access to social protection (health insurance and unemployment benefits)	-0.270**	0.763
Barriers to becoming an entrepreneur—Concerns about workload, working hours and/or responsibilities	-0.258**	0.773
Barriers to becoming an entrepreneur—Unfavourable economic climate	-0.163***	0.850
Important goals—To become wealthy	-0.273*	0.761
Important goals—To create my own job	0.506*	1.659
Important goals—To follow my passion/interest	0.185**	1.204
Initial funding sources—My own savings	0.269*	1.308
Initial funding sources—Family and/or friends	0.207**	1.230
Initial funding sources—Banks	-0.144**	0.866
Initial funding sources—Venture capital investments	0.550*	1.733
Initial funding sources—My current salary	0.360*	1.433
Entrepreneurship training—School or university	-0.178**	0.837
Entrepreneurship training—Entrepreneurship coach or network	0.160**	1.173
Entrepreneurship training—Online content creators (e.g. YouTube, Instagram, TikTok)	0.437*	1.548
Entrepreneurship training—Chamber of commerce	-0.230**	0.794
Entrepreneurship training—EU institutions	0.242**	1.274
Support perceived useful—Support with tax requirements	0.233*	1.262
Support perceived useful—Financial support in the form of grants	0.198**	1.219
Support perceived useful—Microfinance support (e.g. microcredits, microloans, microinsurance)	0.287*	1.332
General opinion—Entrepreneurs only think about profit	-0.185**	0.831
General opinion—Entrepreneurs aim for a better world	0.282**	1.326
Constant	0.310	1.364
*Number of observations*	*5460*	
*Omnibus Tests of Model Coefficients (Chi-square)*	*843*.59*	
*Nagelkerke R Square*	*0*.*214*	
*Hosmer and Lemeshow Test*	*Sig*. *= 0*.*977*	
*Overall percentage of classification table (accuracy)*	*77*.*8%*	

Note

* - 1% significance level

** - 5% significance level

*** - 10% significance level.

Source: Authors estimations.

The level of education significantly influences entrepreneurial intentions. The results indicate that young workers who have completed upper secondary education, whether general or vocational, are less likely to aspire to start their own businesses compared to those who left school before completing lower secondary education. Furthermore, it is noteworthy that individuals with higher education exhibit an even lower propensity towards entrepreneurship.

For this group of young individuals, familiarity with entrepreneurship plays a crucial role. Those who have a clear understanding of what it entails to launch and manage a business are more than three times as likely to express the desire to start their own company. Moreover, the perception that young people are interested in entrepreneurship enhances the likelihood of their own entrepreneurial aspirations, suggesting a potential influence from their social circles. This broader interest in entrepreneurship appears to have a contagion effect, potentially encouraging individuals to personally decide to pursue business opportunities.

The primary motivation for young employees to start their own businesses is the prospect of improved income, followed by the desire for autonomy in selecting work hours and location. These factors are closely associated with a heightened inclination toward entrepreneurship.

Regarding the most important goals, young working individuals who prioritize creating their own jobs and pursuing their passions and interests are more likely to exhibit entrepreneurial intentions. In contrast, those whose primary goal is to become wealthy are less inclined to desire to start their own business. The results show a mix of necessity-driven and opportunity-driven entrepreneurship among young people who work, suggesting that low income and limited autonomy at work, coupled with a desire to pursue personal passions, may drive their intention to start a business. These findings are consistent with other studies [32,36,61].

With respect to the barriers to entrepreneurship, young employees express concerns about several factors: financial risks, access to social protection (such as health insurance and unemployment benefits), workload, working hours, responsibilities, and the overall economic climate. These perceived obstacles all serve as powerful disincentives to young people’s desire to start their own businesses.

The examination of preferred financing sources for potential businesses among young employees revealed a positive association between entrepreneurial intentions and the use of personal savings, salary, funds from family and friends, and venture capital investments. In contrast, a negative correlation was observed between entrepreneurial intentions and the pursuit of initial funding from banks.

The findings also pointed out that young employees favour multiple sources of entrepreneurial training. There is a positive association between the intention to start their own business and the perceived usefulness of entrepreneurial education from EU institutions, entrepreneurship coaches or networks, and online content creators, with the latter generating the strongest influence on entrepreneurial intentions. Once again, formal education provided by schools, universities, or chambers of commerce is not perceived by young individuals as an incentive for entrepreneurship. Those who selected these traditional training options are less likely to express a desire to start a business.

The young individuals who indicated a need for support with tax requirements, financial assistance in the form of grants, or microfinance options (such as microcredits, microloans, and microinsurance) are more likely to exhibit entrepreneurial intentions.

Regarding their overall perception of entrepreneurs, young individuals who believe that entrepreneurs aim to create a better world are more likely to aspire to start their own businesses. Conversely, those who view entrepreneurs as primarily motivated by profit are less likely to pursue entrepreneurial initiatives.

### Young people who study and work

The fourth model is designed to examine the characteristics of young individuals who work and study at the same time, with a focus on their entrepreneurial intentions. The results derived from the estimation of the logistic regression model are presented in Table 6. Young men exhibit a higher propensity for entrepreneurial intentions compared to their female counterparts. Additionally, individuals under the age of 25 demonstrate a greater inclination towards starting their own businesses than those in the 25 to 30 age bracket. Furthermore, young people residing in small or medium-sized towns are more inclined to pursue entrepreneurship than those living in rural areas or villages. The findings align with other research [55], which highlights the importance of the overall level of regional development in shaping the intention to engage in innovative economic activities. One finding that is worth emphasizing is that, for young individuals who are concurrently studying and working, the level of education does not significantly influence their entrepreneurial intentions.

**Table 6:** Estimation results for young people who are still studying and working.

Variable	Coefficient	Odds ratio
Gender (Female—ref)	0.514*	1.671
Age (15–24 years—ref)	-0.347*	0.706
Type of community (Rural area or village—ref)		
*Small or medium sized town*	0.135***	1.145
*Large town/city*	0.107	1.113
Number of children under 15 years (No children—ref)		
*1 child*	0.401*	1.493
*2 children*	0.459*	1.583
*3 or more children*	0.479*	1.614
Current level of education (Lower secondary level—ref)		
*Upper secondary level*, *general education*	0.168	1.183
*Upper secondary level*, *vocational education*	0.189	1.208
*Post‐secondary*, *non‐higher education*	0.251	1.285
*Higher education*	0.122	1.130
Familiarity with entrepreneurship	1.048*	2.853
Young people’s interest in entrepreneurship	0.754*	2.126
Reasons to start own business—Better income prospects	0.169**	1.184
Reasons to start own business—Lack of employment opportunities	0.238**	1.269
Barriers to becoming an entrepreneur—Not enough capital/resources to be self-employed	-0.159**	0.853
Barriers to becoming an entrepreneur—Financial risks involved	-0.274*	0.761
Barriers to becoming an entrepreneur—Unfavourable economic climate	-0.247*	0.781
Important goals—To become wealthy	-0.192*	0.825
Important goals—To create my own job	0.211*	1.235
Important goals—To create jobs for others	0.213**	1.237
Initial funding sources—My own savings	0.180*	1.197
Initial funding sources—Banks	-0.375*	0.687
Initial funding sources—Venture capital investments	0.426*	1.531
Initial funding sources—Business incubator	0.331*	1.392
Initial funding sources—My current salary	0.360*	1.433
Entrepreneurship training—School or university	-0.115***	0.891
Entrepreneurship training—Entrepreneurship coach or network	0.224*	1.251
Entrepreneurship training—Online content creators (e.g. YouTube, Instagram, TikTok)	0.436*	1.547
Entrepreneurship training—Chamber of commerce	-0.173**	0.841
Entrepreneurship training—EU institutions	0.227*	1.255
Support perceived useful—Financial support in the form of conventional loans	0.155***	1.168
Support perceived useful—Education or training	-0.132***	0.876
General opinion—Entrepreneurs create new products and services that benefit us all	0.330*	1.391
General opinion—Entrepreneurs aim for a better world	0.216**	1.241
General opinion—Entrepreneurs are job creators	0.125***	1.134
EU is putting effort into decreasing youth unemployment	-0.114***	0.892
Constant	-0.247	0.781
*Number of observations*	*8117*	
*Omnibus Tests of Model Coefficients (Chi-square)*	*1028*.31*	
*Nagelkerke R Square*	*0*.*194*	
*Hosmer and Lemeshow Test*	*Sig*. *= 0*.*717*	
*Overall percentage of classification table (accuracy)*	*82*.*5%*	

Note

* - 1% significance level

** - 5% significance level

*** - 10% significance level.

Source: Authors estimations.

With regard to family composition, the number of adults in the household is not statistically significant. However, young individuals from households with one or more children are more likely to express entrepreneurial intentions compared to those from childless households. Notably, the probability of aspiring to entrepreneurship increases as the number of children in the household rises.

Familiarity with entrepreneurship, alongside a general interest in the field, emerged, once again, as substantial determinants influencing the intention of young individuals to initiate their own businesses.

The main reasons driving entrepreneurship among young individuals who are both studying and working include a scarcity of employment opportunities and the prospect of enhanced income. These factors are pivotal in propelling their intentions to pursue business intentions. The findings imply that the primary drivers of entrepreneurial intent are linked to addressing gaps in the job market and improving financial stability. This reflects a pragmatic approach to entrepreneurship, seen as a practical solution to existing economic challenges. The findings indicate the prevalence of entrepreneurship by necessity among European youth who work and study at the same time, the results being similar to other theoretical or research papers [32,36,61].

Within this group, the analysis identified several significant barriers to entrepreneurship, including insufficient capital or resources to be self-employed, financial risks, and an adverse economic climate. These factors were found to negatively correlate with the entrepreneurial intentions of the young individuals examined.

The most important goals of young individuals associated with entrepreneurial intentions include creating employment opportunities for themselves and others, which positively correlates with the desire to start a business. Conversely, the aspiration to become wealthy is negatively correlated with the intention to become an entrepreneur, indicating that those focused predominantly on financial success might not be as committed to entrepreneurship or might perceive it as a less viable path compared to other career options. These findings suggest that the motivations driving young people towards entrepreneurship are more aligned with personal fulfilment and societal contribution rather than purely financial gain.

The initial sources of financing that are positively correlated with the entrepreneurial intentions of young individuals who are both studying and working include personal savings, current salary, venture capital investments, and support from business incubators. In contrast, banks are negatively correlated with entrepreneurial intentions, reflecting a general lack of trust among aspiring young entrepreneurs in traditional financial institutions for securing startup capital.

Regarding entrepreneurial training options, young individuals prioritize online content creators on platforms such as YouTube, Instagram, and TikTok, followed by EU institutions and entrepreneurship coaches or networks. Interaction with these learning sources is positively associated with a heightened likelihood of aspiring to start their own businesses. More formal education provided through schools, universities, or chambers of commerce is negatively correlated with the intention to pursue entrepreneurship. These findings indicate that young individuals prefer learning about entrepreneurship from online content creators and practical networks and mentorship, being seen as more relevant and engaging, over formal education.

Young individuals who perceive financial support, such as conventional loans, as beneficial are more likely to demonstrate entrepreneurial intentions. Conversely, those who view education or training as the primary form of support are less inclined to desire to launch their own company, suggesting a preference for financial over educational resources.

Upon examining the general opinion about entrepreneurs of young people who are both working and studying, a positive association was identified between entrepreneurial intentions and the perception that entrepreneurs create new products and services that benefit us all, aim for a better world, and are job creators. The findings suggest that young individuals who hold the view that entrepreneurs contribute positively to society are more likely to be motivated to start their own businesses.

Lastly, young individuals who believe that the European Union is actively working to reduce youth unemployment are less likely to exhibit entrepreneurial intentions. The result indicates that when confidence in institutional efforts to improve labour market conditions is high, the drive to start a new business may diminish. Similar to the young people who are only studying, young people who are studying and working and who have negative attitudes regarding the EU’s effort to reduce employment are more likely to develop their own businesses indicating again the prevalence of the necessity-driven entrepreneurship intentions among the studied sub-groups.

Young people in the European Union who intend to start their own businesses share several common characteristics regardless of their education and employment status. Notably, men under 25 years old and those from larger households with one or more children exhibit a heightened interest in entrepreneurship, thereby validating research hypotheses H_6_ and H_7_. Familiarity with entrepreneurship emerges as a crucial driver of these intentions, validating hypothesis H_5_ for all young people in the European Union. Key motivations for these individuals include creating their own employment, pursuing personal passions, and innovating within their daily activities. Indeed, their primary interests focus on achieving flexibility, adaptability, and independence through their work, characteristics associated with an increased inclination towards entrepreneurial intentions confirming research hypothesis H_2_ regarding the importance of expected outcomes for entrepreneurial decisions. An important source of information for young people seeking entrepreneurship training are the online content creators. Many young individuals turn to platforms such as YouTube, Instagram, and TikTok to gain knowledge about entrepreneurship. This preference for modern tools reflects a broader inclination towards informal learning methods over formal education, as they find traditional schooling demotivating for acquiring entrepreneurial skills. The propensity to save money is prevalent among all young aspiring entrepreneurs and facilitates the process of launching their desired businesses. Generally, those aspiring to start their own business view entrepreneurs positively, seeing them as innovators who create new products and services and contribute to a better world, thereby validating research hypothesis H_1_.

The results also enabled us to identify the specific characteristics and differences among each sub-group. The findings indicate notable variations in the influence of education level on entrepreneurial intentions across different groups. In particular, young individuals currently enrolled in upper secondary education, NEETs who have completed either lower or upper secondary education, as well as employed individuals with lower educational attainment exhibit the highest propensity toward entrepreneurship.

Research hypothesis H_8_, which posits that significant differences in entrepreneurial intentions among EU youth exist based on place of residence, was partially validated. Specifically, NEETs and young people who are both working and studying exhibit a stronger inclination toward entrepreneurship if they reside in small or medium-sized towns rather than in rural areas or villages.

Young people’s motivations for starting their own businesses vary based on their educational background and professional experience. For young NEETs, those who are only working, and those who balance work and study, the primary motivation is the prospect of better income, with these individuals seeking to enhance their financial situation through entrepreneurship. In contrast, young people who are exclusively studying are primarily driven by the desire to seize business opportunities, which they view as the main catalyst for their entrepreneurial intentions.

Young individuals in Europe encounter various barriers to entrepreneurship. For young NEETs, the primary obstacles include a lack of entrepreneurial education, skills, or knowledge, as well as concerns about administrative challenges. This result validates research hypothesis H_3_, but only for this group. The other subgroups of young people face distinct challenges in starting a business. For those who are only working or studying, concerns primarily focus on financial risks and an unfavourable economic climate. Additionally, these individuals show reluctance towards additional working hours and increased responsibilities, even if they would benefit from their efforts. In contrast, young people who balance both work and study face significant difficulties due to insufficient financial resources for self-employment, thereby confirming research hypothesis H_4_.

Young people generally exhibit a reluctance to rely on banks as a source of funding for their entrepreneurial intentions, opting instead for alternative financial resources. Young NEETs only consider personal savings as an initial funding source when starting a business. In contrast, other youth categories are more diverse in their funding approaches, exploring various options such as personal savings, salaries, venture capital, microfinance providers, family and friends, and support from business incubators. Notably, young individuals who balance both studying and working demonstrate a significant diversification of their funding sources, drawing on their combined theoretical and practical knowledge, and opting for resources ranging from salaries to support from business incubators.

## Conclusions

Young Europeans are comparatively more vulnerable in the labour market than other age groups, which leads them to look for job alternatives like entrepreneurship. While it may not be a miracle solution, youth entrepreneurship can facilitate their transition into well-paying employment. This study examined the entrepreneurial intentions of young Europeans, using data from the Flash Eurobarometer 513 –Social Entrepreneurship and Youth. The research specifically focused on the characteristics of four distinct groups of young individuals based on their educational and employment statuses.

The profile of young NEETs who aspire to be entrepreneurs is characterized as follows: predominantly men, under 25 years of age, with an average level of education, residing in small or medium-sized cities, and from households with one or two children. Their motivations for becoming entrepreneurs include creating their own jobs, following their passions and interests, and implementing their innovative ideas. They do not necessarily believe that entrepreneurs earn substantial amounts of money, although they acknowledge better income prospects. They express concerns about the lack of knowledge and skills required to start and manage a business and prefer to learn about entrepreneurship through online platforms rather than from formal education. Additionally, they would require support for business plan development and they intend to use their own savings as the primary source of financing for their business.

Young individuals still studying who aspire to start their own businesses are predominantly male and are more likely to come from households with one child. They are typically enrolled in upper secondary education, whether general or vocational, and exhibit a strong familiarity with entrepreneurship, which is a key driver of their entrepreneurial intentions. They are motivated by the desire to create their own employment opportunities and implement innovative ideas. Preference for diverse financing sources, such as personal savings, microfinance, and venture capital is evident, with a notable skepticism toward traditional banks. Their entrepreneurial goals are further supported by learning from online content creators and engagement with entrepreneurship coaches or networks, whereas formal educational settings do not favour entrepreneurship. They view entrepreneurs as creators of valuable innovations who aim to improve the world and achieve significant financial success. Additionally, those who believe that the EU is effectively addressing youth unemployment are less likely to pursue entrepreneurship, suggesting a tendency towards traditional employment when confidence in institutional efforts is high.

The profile of young working individuals with entrepreneurial intentions includes young men, especially those under 25 years old. Household composition matters, with those from households with two or more children being more likely to pursue entrepreneurship. Education level influences their intentions, with those having lower secondary education showing higher entrepreneurial aspirations than those with upper secondary or higher education. Familiarity with entrepreneurship and a perception that peers are interested in entrepreneurship significantly boost their intentions. Key motivations are better income prospects and autonomy, while financial risks, lack of social protection, and unfavourable economic climate are major barriers. Preferred financing sources are personal savings, salaries, funds from family and friends, and venture capital, while bank financing is avoided. Entrepreneurial training from EU institutions, entrepreneurship coaches, networks, and online content creators is valued over formal education. Support with tax requirements, grants, or microfinance options increases their entrepreneurial intentions, and they are more likely to aspire to entrepreneurship if they believe entrepreneurs aim to create a better world rather than focusing solely on profit.

Young individuals who are both studying and working and manifest entrepreneurial intentions are typically male, under 25 years old, and from small or medium-sized towns. Their entrepreneurial drive is not influenced by education level. They come from households with one or more children, with entrepreneurial intentions increasing with the number of children. Key motivators include a lack of job opportunities and the prospect of higher income. Familiarity with entrepreneurship and interest in the field are crucial determinants. Barriers include insufficient capital, financial risks, and a poor economic climate. Preferred financing sources are personal savings, current salary, venture capital, and business incubators, while traditional banks are not trusted. They prefer entrepreneurial training from online content creators and networks rather than formal education. Viewing financial support as beneficial, rather than education or training is linked to higher entrepreneurial intentions. Additionally, those who believe the EU is actively reducing youth unemployment are less likely to pursue entrepreneurship.

The study identified several key common trends among young aspiring entrepreneurs in the European Union. Men under 25 years old and those from larger households show a greater interest in entrepreneurship. Familiarity with entrepreneurship is a major driver and young people prefer online content creators for entrepreneurship training over traditional education. The analysis also highlighted notable differences among the four groups studied. NEETs and working individuals are primarily motivated by the prospect of better income, while students are driven by business opportunities. Barriers to entrepreneurship include insufficient education for NEETs and financial risks for the other groups. Funding preferences vary: NEETs rely mainly on personal savings, whereas those who balance studying and working explore a diverse range of sources, including venture capital and support from business incubators.

The study’s findings suggest several key political implications for fostering youth entrepreneurship in the European Union. First, there is a need to enhance educational programs by emphasizing practical entrepreneurial skills and incorporating informal learning methods that align with the preferences of young individuals. Policymakers should consider developing targeted initiatives for NEETs and young people in diverse educational and professional situations, addressing gaps in entrepreneurial education and providing tailored support. Additionally, regional development policies should be designed to promote entrepreneurship in less developed areas, improving access to resources and financial support. The preference for alternative funding sources, such as venture capital and business incubators, highlights the need for policies that facilitate access to diverse financing options. Addressing these factors can help create a more supportive environment for young entrepreneurs and mitigate barriers related to financial risks and economic conditions.

This study offers a novel perspective by comparing European youth based on their educational and employment status, identifying the entrepreneurial drivers among NEETs, young workers, students, and those who both work and study. Nevertheless, the research has certain limitations, particularly related to data availability. For instance, we were unable to include variables such as country-level institutions, which are essential for a deeper understanding of youth entrepreneurial intentions [49]. Additionally, the study relies on cross-sectional data, providing only a snapshot of youth intentions at a single point in time. Furthermore, the study focuses solely on the intentions of young people to become entrepreneurs, without examining whether these intentions lead to the actual creation of businesses. Finally, another limitation of this study is the potential for endogeneity, as we are using variables that capture subjective opinions, such as reasons for starting a business, perceived barriers, important goals, or expected results of entrepreneurial initiatives. These subjective measures may be influenced by reverse causality, as entrepreneurial intentions could shape individuals’ perceptions of entrepreneurship. Additionally, unobserved characteristics, such as personal traits or risk tolerance, could simultaneously affect both the subjective opinions and the outcome, leading to omitted variable bias. Moreover, the potential for measurement error in self-reported data may bias the estimates[62]. While efforts were made to minimize these issues through careful variable selection and model specification, future research may address endogeneity more comprehensively.

## Supporting information

S1 Table Chi-square test for the association between variables. (DOCX)
