# Supplementary material for: Exploring entrepreneurial intention among European Union youth by education and employment status
Source: PLoS One. 2025 Jan 24;20(1):e0318001. doi: 10.1371/journal.pone.0318001 (PMC11761609; doi:10.1371/journal.pone.0318001)
Supplement: S1 Table — (DOCX) [file pone.0318001.s001.docx]

**S1 Table. Chi-square test for the association between variables**

| **Variables** | Entrepreneurship intention (Would you consider setting up your own business?) | | | | |
| --- | --- | --- | --- | --- | --- |
|  | **Young NEETs**  **(sig.)** | **Young people who are only studying**  **(sig.)** | **Young people who are only working**  **(sig.)** | **Young people who are both studying and working**  **(sig.)** |  |
| Gender | **<0.001** | **<0.001** | **<0.001** | **<0.001** |  |
| Age of the respondent | **<0.001** | **0.027** | **<0.001** | **<0.001** |  |
| Type of community | **<0.001** | **<0.001** | 0.060 | **<0.001** |  |
| Household size, 15+ | 0.064 | **0.012** | 0.210 | **<0.001** |  |
| Number of children under 15 years | 0.072 | **<0.001** | **<0.001** | **<0.001** |  |
| Level of education | 0.217 | - | **<0.001** | - |  |
| Familiarity with entrepreneurship | **<0.001** | **<0.001** | **<0.001** | **<0.001** |  |
| Young people's interest in entrepreneurship | **<0.001** | **<0.001** | **<0.001** | **<0.001** |  |
| - Entrepreneurs create new products and services that benefit us all - Entrepreneurs only think about profit - Entrepreneurs aim for a better world - Entrepreneurs make a lot of money - Entrepreneurs are job creators | **<0.001**  **0.013**  0.326  **0.019**  **0.001** | **<0.001**  **0.025**  **<0.001**  **<0.001**  **<0.001** | **0.002**  **<0.001**  **<0.001**  0.051  **<0.001** | **<0.001**  **<0.001**  **<0.001**  **0.001**  **<0.001** |  |
| - Freedom to choose time and place of work - Better income prospects - Exploiting a business opportunity - Lack of employment opportunities - To become wealthy - To create my own job - To follow my passion/interest - To create jobs for others - To put innovative ideas into practice - Financial risks involved - Access to social protection (health insurance and unemployment benefits) - Concerns about red tape and administrative issues (e.g. accounting obligations, tax returns) - Concerns about workload, working hours and/or responsibilities - Unfavourable economic climate | **0.001**  **<0.001**  0.432  **0.100**  **0.005**  **<0.001**  **<0.001**  **0.003**  **0.003**  **0.002**  0.204  **0.002**  0.678  **<0.001** | **<0.001**  **<0.001**  0.044  0.058  **<0.001**  **<0.001**  **<0.001**  **0.595**  **<0.001**  **<0.001**  **<0.001**  **0.019**  **0.035**  **<0.001** | **<0.001**  **<0.001**  0.764  0.762  0.045  **<0.001**  **<0.001**  **<0.001**  0.060  **<0.001**  **<0.001**  0.149  **0.011**  **0.006** | **<0.001**  **<0.001**  **0.004**  0.117  **0.001**  **<0.001**  **<0.001**  **<0.001**  **<0.001**  **<0.001**  **<0.001**  **<0.001**  **0.040**  **<0.001** |  |
| - Lack of knowledge/education/skills on how to start and run a business - Support with developing the business plan - Support with tax requirements - Education or training | **0.007**  **<0.001**  **0.024**  **0.003** | **<0.001**  0.064  **0.007**  **<0.001** | **<0.001**  **0.015**  **0.002**  **<0.001** | **<0.001**  **<0.001**  **0.003**  **<0.001** |  |
| - Not enough capital/resources to be self-employed - Financial support in the form of grants - Financial support in the form of conventional loans - Microfinance support (e.g. microcredits, microloans, microinsurance) | **<0.001**  0.292  0.224  **<0.001** | **<0.001**  **<0.001**  **0.006**  **0.002** | **<0.001**  **<0.001**  0.263  **0.014** | **<0.001**  **<0.001**  **0.004**  **<0.001** |  |
| - My own savings - Family and/or friends - Banks - Microfinance providers - Venture capital investments - Business incubator - My current salary | **<0.001**  **<0.001**  **<0.001**  0.034  **<0.001**  0.425  **<0.001** | **<0.001**  **<0.001**  **<0.001**  **0.032**  **<0.001**  0.465  **<0.001** | **<0.001**  **<0.001**  **<0.001**  **0.016**  **<0.001**  0.305  **<0.001** | **<0.001**  **0.002**  **<0.001**  **<0.001**  **<0.001**  **<0.001**  **<0.001** |  |
| - School or university - Entrepreneurship coach or network - Online content creators (e.g. YouTube, Instagram, TikTok) - Chamber of commerce - Job centre - EU institutions | **0.017**  **0.019**  **<0.001**  0.257  0.897  0.343 | **<0.001**  **<0.001**  **<0.001**  0.041  **0.001**  0.495 | **<0.001**  **<0.001**  **<0.001**  0.051  **0.022**  **0.004** | **<0.001**  **<0.001**  **<0.001**  **0.013**  0.735  **<0.001** |  |
| How much effort do you feel the European Union is putting into decreasing youth unemployment? | **0.004** | **0.044** | **0.029** | **<0.001** |  |

Source: Authors’ calculations
